# Circulating tumor cell copy-number heterogeneity in *ALK*-rearranged non-small-cell lung cancer resistant to ALK inhibitors

**DOI:** 10.1038/s41698-021-00203-1

**Published:** 2021-07-16

**Authors:** Marianne Oulhen, Patrycja Pawlikowska, Tala Tayoun, Marianna Garonzi, Genny Buson, Claudio Forcato, Nicolò Manaresi, Agathe Aberlenc, Laura Mezquita, Yann Lecluse, Pernelle Lavaud, Charles Naltet, David Planchard, Benjamin Besse, Françoise Farace

**Affiliations:** 1 Gustave Roussy, Université Paris-Saclay, “Rare Circulating Cells” Translational Platform, CNRS UMS3655 – INSERM US23 AMMICA, VILLEJUIF, France; 2 INSERM, U981 “Identification of Molecular Predictors and new Targets for Cancer Treatment”, VILLEJUIF, France; 3 Univ Paris Sud, Université Paris-Saclay, Faculty of Medicine, LE KREMLIN-BICETRE, France; 4 Menarini Silicon Biosystems S.p.A, BOLOGNA, Italy; 5 Gustave Roussy, Université Paris-Saclay, Department of Medicine, VILLEJUIF, France; 6 Gustave Roussy, Université Paris-Saclay, “Flow cytometry and Imaging” Platform, CNRS UMS3655 – INSERM US23AMMICA, VILLEJUIF, France

**Keywords:** Cancer, Molecular medicine

## Abstract

Gatekeeper mutations are identified in only 50% of the cases at resistance to *Anaplastic Lymphoma Kinase* (*ALK*)-tyrosine kinase inhibitors (TKIs). Circulating tumor cells (CTCs) are relevant tools to identify additional resistance mechanisms and can be sequenced at the single-cell level. Here, we provide in-depth investigation of copy number alteration (CNA) heterogeneity in phenotypically characterized CTCs at resistance to ALK-TKIs in *ALK*-positive non-small cell lung cancer. Single CTC isolation and phenotyping were performed by DEPArray or fluorescence-activated cell sorting following enrichment and immunofluorescence staining (ALK/cytokeratins/CD45/Hoechst). CNA heterogeneity was evaluated in six *ALK*-rearranged patients harboring ≥ 10 CTCs/20 mL blood at resistance to 1^st^ and 3^rd^ ALK-TKIs and one presented gatekeeper mutations. Out of 82 CTCs isolated by FACS, 30 (37%) were ALK^+^/cytokeratins^-^, 46 (56%) ALK^-^/cytokeratins^+^ and 4 (5%) ALK^+^/cytokeratins^+^. Sequencing of 43 CTCs showed highly altered CNA profiles and high levels of chromosomal instability (CIN). Half of CTCs displayed a ploidy >2n and 32% experienced whole-genome doubling. Hierarchical clustering showed significant intra-patient and wide inter-patient CTC diversity. Classification of 121 oncogenic drivers revealed the predominant activation of cell cycle and DNA repair pathways and of RTK/RAS and PI3K to a lower frequency. CTCs showed wide CNA heterogeneity and elevated CIN at resistance to ALK-TKIs. The emergence of epithelial *ALK*-negative CTCs may drive resistance through activation of bypass signaling pathways, while *ALK*-rearranged CTCs showed epithelial-to-mesenchymal transition characteristics potentially contributing to ALK-TKI resistance. Comprehensive analysis of CTCs could be of great help to clinicians for precision medicine and resistance to ALK-targeted therapies.

## Introduction

Molecularly targeted therapies have produced substantial clinical benefits in about 20% of non-small cell lung cancer (NSCLC) patients, including a 4% subgroup harboring the *anaplastic lymphoma kinase* (*ALK*) gene rearrangements^1^. In 2012, the tyrosine kinase inhibitor (TKI) crizotinib obtained Food and Drug Administration (FDA) approval and became a standard therapy in patients with advanced *ALK-*rearranged NSCLC. Studies have shown improvement in response rate and progression-free survival (PFS) compared with chemotherapy^2^. For patients relapsing on crizotinib, more potent and selective second-generation ALK inhibitors such as ceritinib, alectinib and brigatinib, have produced substantial clinical benefits, re-inducing responses to treatment in the majority of crizotinib-resistant patients^3–5^. Mutations in ALK kinase domain emerging on treatment with second-generation ALK inhibitors are in most cases targetable by the third-generation TKI lorlatinib^1,6^. Sequential therapy with increasing potent inhibitors targeting a spectrum of distinct secondary *ALK* resistance mutations is now the standard of care for *ALK*-rearranged NSCLC.

Despite clinical benefit of ALK-TKIs, patients ultimately progress. Several mechanisms of acquired resistance have been described including gatekeeper mutations within the ALK tyrosine kinase domain, gain of *ALK* native copies, activation of by-pass parallel or downstream ALK-independent signaling pathways and lineage transdifferentiation^7^. A minority of crizotinib-resistant patients acquire secondary *ALK* mutations while most of them develop resistance through activation of bypass pathways such as somatic mutations in PI3K/RTK/RAS or gene amplification of *EGFR*, *cKIT*, *KRAS,* or *MET*^7–10^. Next-generation ALK-TKIs have shown differential sensitivity profiles with respect to ALK kinase domain mutations. Various alternative oncogenic drivers and bypass non-mutation-driven pathways have also emerged as an important resistance mechanism to next-generation ALK-TKIs, which deserves more in-depth investigation^9,10^. Identifying these mechanisms by comprehensive methods is essential to devise new therapeutic strategies to overcome drug resistance.

Phenotypes associated with treatment resistance require complex interaction between processes, among which genomic instability is a driving force promoting continuous modification of tumor genomes, leading to tumor heterogeneity and clonal evolution. Chromosomal instability (CIN) results from the occurrence and tolerance of chromosome segregation errors during cell division and contributes to tumor heterogeneity. CIN leads to copy number alteration (CNA) heterogeneity and creates a pool of genetically diverse cells that could be subjected to selection. CNAs are prevalent in a vast majority of cancers and CNA patterns—such as gains of oncogenes or losses of tumor suppressors—have been suggested as an acquired resistance mechanism in different tumors, including NSCLC^11^. Large-scale alterations in ploidy are also a consequence of CIN and whole-genome doubling (WGD) has been shown to accelerate cancer evolution^12^. CIN is also associated with an increased risk of recurrence or death in NSCLC, supporting its potential value as a prognostic factor in tumor biopsies and circulating tumor DNA (ctDNA)^13^.

At disease progression on ALK-TKIs, genomic re-profiling of tumor biopsies is needed to portray the full spectrum of drug resistance and identify resistant heterogeneous tumor cell subpopulations. However, tumor biopsy feasibility is limited to accessible sites and provides low quantity of tumor tissue that may underestimate the complexity of the tumor genomic landscape. Non-invasive molecular analysis of liquid biopsy components is actively evaluated to overcome the limitations of tissue sequencing. Circulating tumor cells (CTCs) are likely released from spatially distinct metastatic sites and may contain clones with tumorigenic activity^14^. CTC analysis may therefore provide a comprehensive genomic profile of the tumor and, most importantly, of cell clones relevant for metastatic activity and disease progression^15^. The prognostic and pharmacodynamic biomarker values of CTC counts have been reported in numerous solid tumors^16–18^. Moreover, a plethora of CTC technologies have been developed to identify molecular biomarkers predictive of sensitivity or resistance for therapy selection^15,19,20^. In NSCLC, we and others have reported the ability to detect *ALK*-rearrangement in CTCs from *ALK*-rearranged NSCLC patients^19,21^. We have also identified CTCs harboring a high level of numerical CIN in *ROS1*-rearranged NSCLC^22^ and reported the predictive value of CTCs with aberrant *ALK* copy number for crizotinib efficacy^23^. More recently, a few studies have exploited single CTC profiling to investigate therapeutic resistance mechanisms and underlying tumor genomic heterogeneity^24–27^. We have performed a single CTC sequencing workflow in an exploratory study of 17 *ALK*-rearranged crizotinib- or lorlatinib-resistant patients, which allowed us to identify heterogeneous resistance mutations to ALK-TKIs in both ALK kinase domain and alternative bypass pathways^27^.

Herein, we selected six of these 17 *ALK*-rearranged patients harboring high CTC levels (≥10 CTCs/20 mL blood) at resistance to ALK-TKIs and provided a comprehensive description of CTC CNA profiles according to epithelial and/or ALK expression characteristics. We characterized CNA heterogeneity in CTCs through assessment of their CIN and hierarchical clustering and identified CNA drivers in numerous ALK-independent pathways. Our results highlight the potential utility of CNA investigation in single CTCs to portray the heterogeneous resistance landscape to ALK-TKIs and identify potentially targetable alternate bypass pathways in *ALK*-positive NSCLC patients.

## Results

### Clinical characteristics of patients

Six *ALK*-rearranged NSCLC patients (P41, P43, P45, P46, P49, and P50) with CTC counts ≥10 CTCs/20 mL blood at resistance to ALK-TKI were analyzed in this study. Patients’ clinicobiological characteristics are presented in Table 1. All tumors were adenocarcinoma. Three patients were nonsmokers. Blood samplings for CTC analysis were performed at radiological disease progression on ALK-TKIs. Four patients (P43, P46, P49, and P50) progressed on first-line crizotinib and two patients (P41, P45) on lorlatinib as second-line or beyond. P41 received crizotinib for 10 months and ceritinib for 16 months before lorlatinib. P45 received crizotinib for 9.5 months, ceritinib for 1.5 months and brigatinib for 3.5 months before lorlatinib. P43, P46, P49, and P50 PFS on crizotinib were 4.7, 13.7, 3.6, and 9.3 months respectively. P41 and P45 PFS on lorlatinib were 13.3 and 3.6 months respectively. As previously reported, resistance mutations have been examined in single CTCs of three patients (P43, P45, and P49)^27^. Two compound mutations (*ALK*^G1202R/F1174C^, *ALK*^G1202R/T1151M^) were identified in single CTCs of P45 including one (*ALK*^G1202R/F1174C^) also detected in the corresponding tumor biopsy.

**Table 1:** Clinicobiological characteristics of *ALK*-rearranged NSCLC patients.

Patient	Treatment	Age at baseline (y/o)	Sex	Smoking status (PY)	Numbers of metastatic sites at baseline	ECOG PS at baseline	Number of previous treatment lines	Number of previous treatment lines with prior ALK inhibitor	Duration of treatment (months)	Progression-free survival (months)	Count of CTCs by CellSearch at PD (/7.5 mL)	Count of CTCs by FACS at PD (/20 mL)	Biopsy
P43	Crizotinib	68	F	0	2	1	0	0	4.7	4.7	0	15	No
P46	Crizotinib	29	M	5	3	0	0	0	13.7	13.7	0	12	No
P49	Crizotinib	59	F	20–25	3	1	3	0	3.6	3.6	13	24	No
P50	Crizotinib	69	M	40	2	2	1	0	9.9	9.3	7	10	No
P41	Lorlatinib	42	M	0	2	0	3	2	13.5	13.3	0	10	No
P45	Lorlatinib	59	F	0	4	2	3	3	4.1	3.6	2	11	Post-progression

*ECOG* Eastern Cooperative Oncology Group, *PS* performance status, *PY* pack-year, *y/o* years old, *PD* progression disease.

### Phenotypical heterogeneity of CTCs

**Fig. 1:**
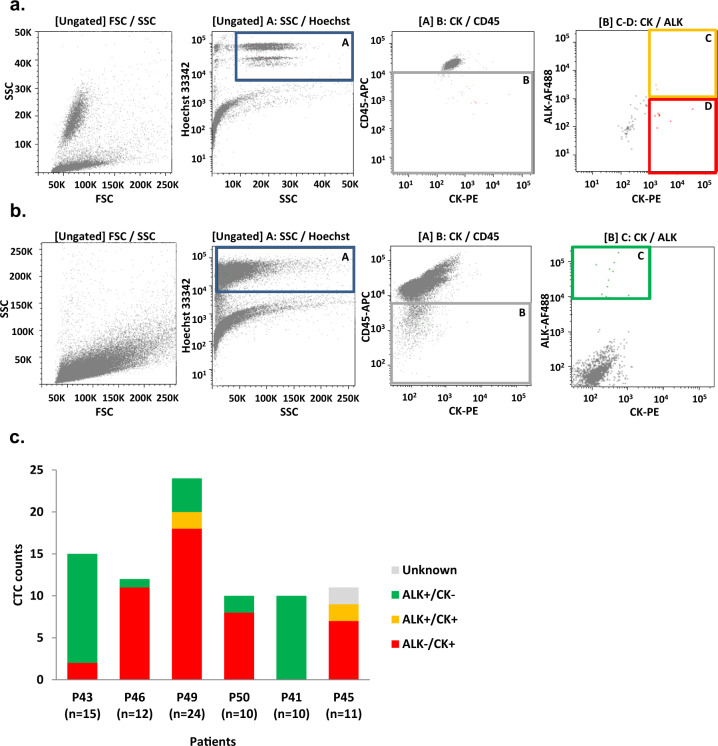
Phenotypic analysis of single CTCs from ALK-TKI resistant patients according to cytokeratins and ALK expression. **a** Representative FACS plots (P45) with applied gating for ALK^+^/CK^+^ (orange) and ALK^−^/CK^+^ (red) CTCs. Hoechst-positive elements were selected using the gate A. The second gate enabled selecting CD45-APC-negative events (gate B). Two populations including Hoechst^+^/CD45-APC^−^/CK-PE^+^/ALK-AF488^+^ and Hoechst^+^/CD45-APC^−^/CK-PE^+^/ALK-AF488^−^ CTCs were identified using gates C and D respectively. **b** Representative FACS plots (P41) with applied gating for ALK^+^/CK^−^ (green) CTCs. Hoechst-positive elements were selected using the gate A. The second gate enabled selecting CD45-APC-negative events (gate B). The Hoechst^+^/CD45-APC^−^/CK-PE^−^/ALK-AF488^+^ CTC population was identified using gate C. **c** Distribution of CTC sub-populations in patients resistant to ALK-TKIs. Note that *ALK*^G1202R/F1174C^ and *ALK*^G1202R/T1151M^ compound mutations were previously identified in one ALK^−^/CK^+^ and one unknown phenotype CTC of P45 respectively. A *TP53*^E286^ mutation was previously identified in one P43 ALK^+^/CK^−^ CTC^27^.

According to a previously reported workflow^27^, CTCs were detected in 20 mL blood samples by combining hematopoietic cell depletion, four-color immunofluorescent staining (ALK/CK/CD45/Hoechst), and FACS. Three different CTC phenotypes were observed: epithelial CTCs expressing only CK (ALK^−^/CK^+^) (Fig. 1a, red square), CTCs expressing both CK and ALK markers (ALK^+^/CK^+^) (Fig. 1a, orange square) and CTCs expressing only ALK (ALK^+^/CK^−^) (Fig. 1b). Eighty-two CTC samples were detected and isolated by FACS. The number of CTCs (median 11.5, range [10–24]) and their phenotypic distribution per patient are shown in Fig. 1c. ALK^+^ (i.e., *ALK*-rearranged)/CK^−^ CTCs represented 37% (30/82) of total CTCs. ALK^−^/CK^+^ epithelial CTCs represented 56% (46/82) while double-positive ALK^+^/CK^+^ CTCs represented 5% (4/82). CTC phenotypes were relatively homogeneous for each patient. In patients P43 and P41, almost all CTCs were *ALK*-rearranged with no CK expression, while in the four remaining patients (P46, P49, P50, P45) the vast majority of CTCs were epithelial and did not harbor *ALK*-rearrangement. In parallel, the CellSearch system detected 13, 7, and 2 CTCs per 7.5 mL blood in P49, P50, and P45 respectively (Table 1). Six and two CellSearch CTCs were isolated from patients P49 and P50 respectively using the DEPArray technique (Supplementary Fig. 1). Using the two CTC isolation techniques, a total of 90 CTC samples were obtained. Considering all the samples including CTCs, corresponding controls (germline DNA and leucocytes) and the tumor biopsy (P45), 52% (54/104) of samples showed high-quality Genome Integrity Index (GII) of 3 or 4. We further included three samples with lower GII (2 or 1) to increase the data points on selected patients. In fact, low-pass whole-genome sequencing (LP-WGS) is relatively tolerant to lower WGA quality. The above numbers take into account as CTCs only those falling into the refined gating for FACS sorting which was applied as a feedback from LP-WGS analysis itself. Overall, including the leukocyte control for each patient, one biopsy, and single-cells initially classified as putative CTCs and then discarded after refining FACS gating, a total of 83 samples underwent LP-WGS and 92% (76/83) passed the sequencing quality controls. 43 CTC samples in total were further analyzed (Supplementary Table 2).

### CNA heterogeneity in CTCs

**Fig. 2:**
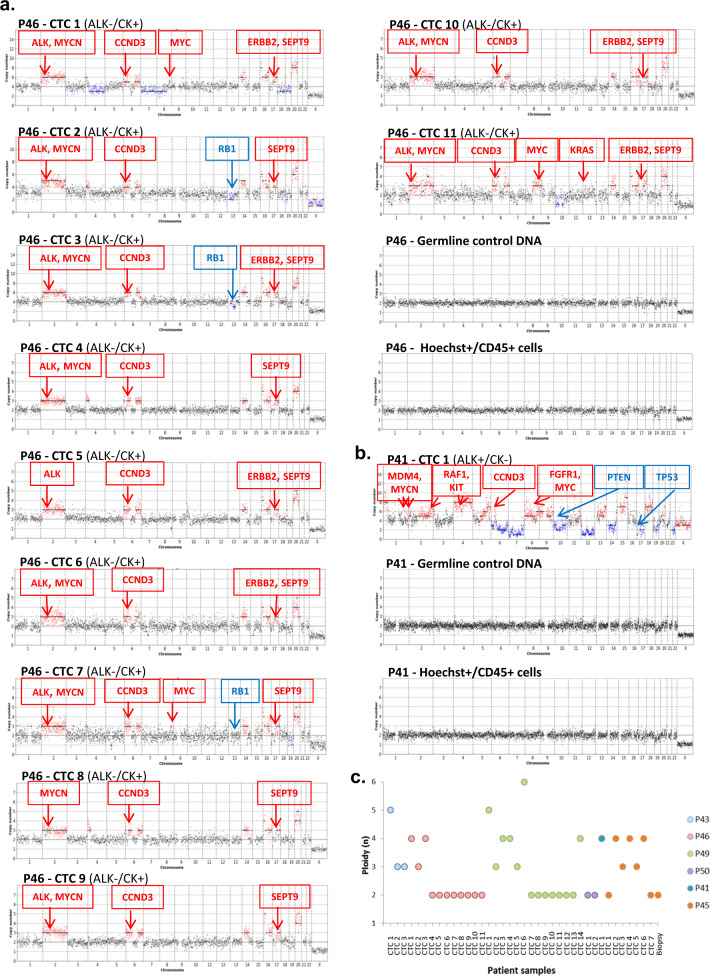
Low-pass whole-genome CNA profiles and ploidy of CTCs. CNAs in predominant pathways are annotated (gain in red, loss in blue). **a** CNA profiles of CTCs, corresponding leucocytes, and germline DNA from patient P46 resistant to crizotinib. **b** CNA profile of CTC, corresponding leucocytes, and germline DNA from patient P41 resistant to lorlatinib. **c** Ploidy determined for each CTC sample. P43 samples contain pools of 5 (CTC 1) or 10 (CTC 2, CTC 3) CTCs. CTC 1 of P46 is a pool of 2 CTCs. All other samples are single CTCs.

The detailed list of CNAs is presented in Supplementary Table 1. CNA profiles were generated from the 43 CTC samples (Fig. 2, Supplementary Fig. 2). Five single ALK^−^/CK^+^ cells displayed flat CNA profiles comparable to WBC controls and were likely to be normal epithelial cells as reported by other groups^28^ (Supplementary Fig. 2e). Twenty-seven single CTCs (P46, P49, P50) and 4 CTC pools (CTCs 1, 2, and 3 from P43, CTC 1 from P46) from the four crizotinib-resistant patients displayed highly altered copy numbers and showed high inter-patient diversity of their CNA profiles. Furthermore, a variable degree of intra-patient heterogeneity was observed. All ALK^−^/CK^+^ CTCs from P46 showed similar alterations in regions of chromosome 2, including gains of *ALK* and *MYCN* genes, alterations in chromosomes 6 and 17 including *CCND3* and *SEPT9* gains, as well as various alterations in other chromosomal regions (Fig. 2a). All four non-epithelial *ALK*-rearranged (ALK^+^/CK^−^) CTCs from P43 and P41 presented aberrant CNA profiles (Supplementary Fig. 2a, b). The three ALK^+^/CK^−^ CTCs from P43 presented distinct CNA profiles. P49 and P50 ALK^−^/CK^+^ CTCs also harbored highly altered copy number patterns (Supplementary Fig. 2b, c). Gains in chromosome 8, such as *FGFR1* or *MYC* genes, and losses in chromosomes 13 such as *RB1* were observed in CTCs 1, 3, 6, 8, 9, 11, and 12 from P49. Heterogeneous CNA patterns were also observed in CTCs from the two patients relapsing on lorlatinib. Five out of seven P45 CTCs (CTCs 1, 2, 4, 6, and 7) presented highly altered profiles (Supplementary Fig. 2d). Important intra-patient diversity was observed among the seven CTCs of P45 while fewer altered regions were detected in the CNA profile of the corresponding tumor biopsy (Supplementary Fig. 2d). Overall, both *ALK*-rearranged non-epithelial (ALK^+^/CK^−^) and epithelial CTCs without *ALK*-rearrangement (ALK^−^/CK^+^) harbored highly altered and diverse CNA profiles. Important inter-patient heterogeneity is observed at resistance to ALK-TKI, suggesting that none of the chromosomal regions were particularly prone to CNA in CTCs post-TKI treatment. Moreover, variable degrees of intra-patient tumor diversity were observed, except in P46 epithelial CTCs which harbored recurrent altered regions such as chromosome 2 (*ALK* and *MYCN* gains), chromosome 6 (*CCND3* gain), and chromosome 17 (*SEPT9* gain).

### Chromosomal instability in CTCs

Ploidy level estimation was performed on the 38 CTC samples with aberrant CNA profiles (Fig. 2c). 19 CTC samples (50%) harbored a ploidy above 2n. Interestingly, all four *ALK*-rearranged non-epithelial (ALK^+^/CK^−^) CTCs had a ≥3n ploidy. Tetraploidy was observed in epithelial CTCs 1 and 3 from P46 and in CTCs 3, 4, and 14 from P49. Half of the epithelial CTCs from P49 presented increased ploidy (≥3n) and CTC 6 displayed a hexaploid content (6n). CTCs with ≥4n possibly experienced WGD, which is considered an early event in cancer evolution and a predictive factor of poor prognosis in many tumor types, including NSCLC^13^. Interestingly, increased ploidy was observed in epithelial CTCs from lorlatinib-resistant patients, with six out of eight single CTCs displaying ≥3n. Four epithelial CTCs with WGD were identified (CTC 1 from P41 and CTCs 2, 4, 6 from P45). These data show important heterogeneity in DNA ploidy in CTCs of ALK-TKI-resistant patients irrespective of their phenotypic characteristics, which may impact CIN and consequently tumor evolution. CTCs displayed a remarkable CIN at resistance to TKIs, which was even more pronounced in samples that acquired WGD (≥4n).

### Hierarchical clustering

**Fig. 3:**
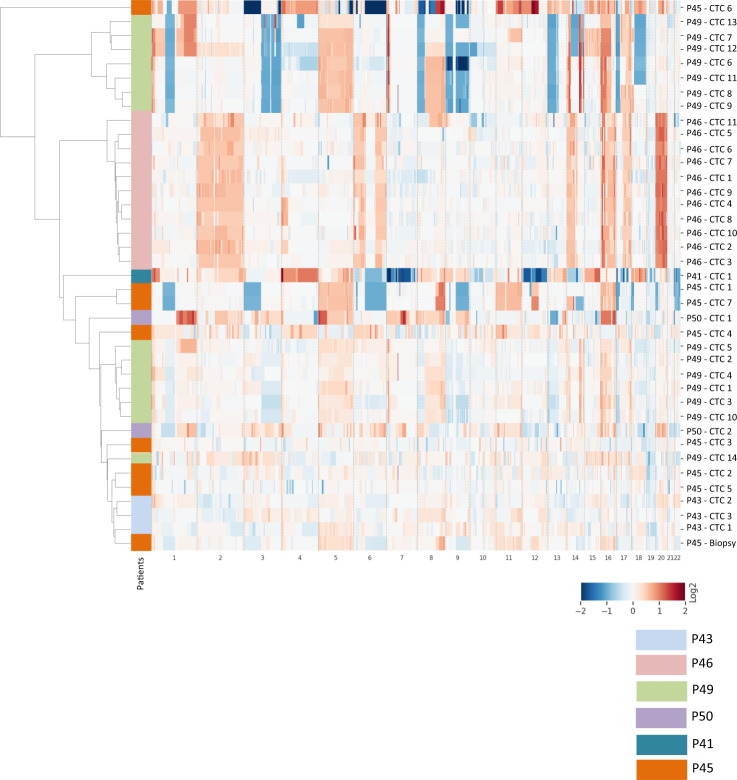
Hierarchical clustering of CTCs according to low-pass whole-genome CNA from crizotinib-resistant (P43, P46, P49, P50) and lorlatinib-resistant (P41, P45) patients. The heatmap shows the log2 values between −2 and +2 with positive values in red (gain) and negative values in blue (loss) respectively to the main ploidy.

We performed hierarchical clustering to distinctly identify recurrently altered chromosomal regions and evaluate intra- and inter-patient similarity in CTC samples (Fig. 3). ALK^+^/CK^−^ CTCs and ALK^−^/CK^+^ CTCs from crizotinib-resistant patients P43 and P46 respectively formed two distant and independent clusters. CK^+^ CTCs of crizotinib-resistant patient P49 was split into two distant clusters containing 7 and 6 CTCs respectively, while the two ALK^−^/CK^+^ CTCs from P50 clustered separately. The seven (mainly CK^+^) CTCs from lorlatinib-resistant patient P45 also clustered separately and were more or less distant from the corresponding tumor biopsy. Hierarchical clustering confirmed high inter-patient diversity at resistance to crizotinib. Despite differences in CNA profiles and ploidy, some intra-patient similarity was observed reflecting a common aberrant genomic background. Furthermore, data also highlights significant intra-patient genomic diversity in P45 CTCs at resistance to lorlatinib.

### CNA driver classification in signaling pathways

**Fig. 4:**
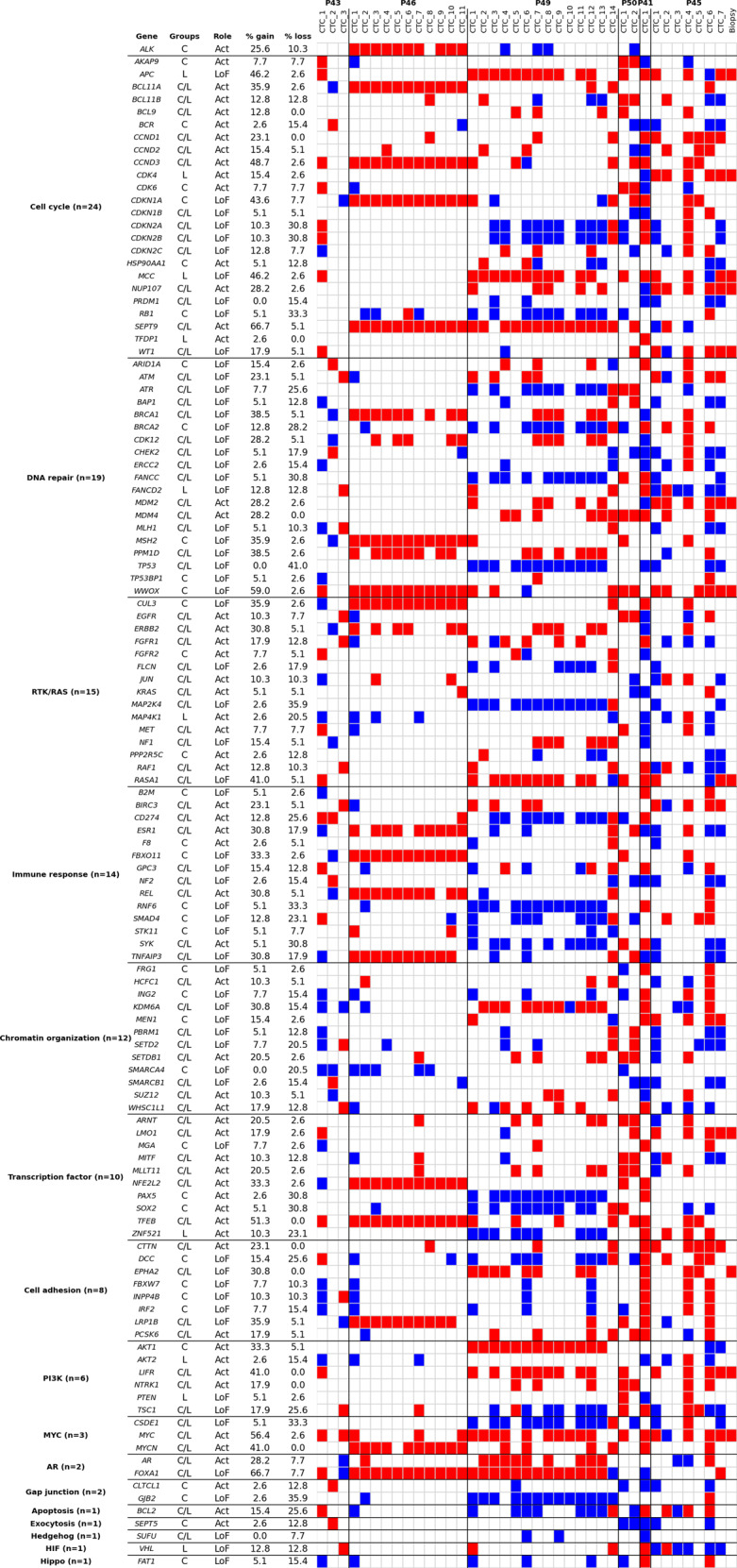
Heatmap of CNA oncogenic drivers in CTC samples. The altered genes are attributed to pathways. The pathways are sorted from the most to the least altered. The number of altered genes per pathway is shown in parentheses. CNA drivers present in patients at resistance to crizotinib only, in patients at resistance to lorlatinib only, and in both, are annotated “C”, “L” and “C/L” respectively in the “Groups” column. CNA driver function (activating or loss of function) is mentioned in the “Role” column. Frequencies of gain and loss in the 39 samples (38 CTC samples and 1 biopsy) are provided. Red and blue colors represent gains and loss respectively.

We then focused on CNAs with oncogenic driver activity. A total of 121 CNA drivers, known or predicted by Cancer Genome Interpreter (CGI), were identified in gain or loss regions across the 43 analyzed CTCs. 37/121 (30.6%) CNA drivers were present exclusively in crizotinib-resistant patients, 10/121 (8.3%) in lorlatinib-resistant patients and 74/121 (61.2%) were shared by both groups (Supplementary Fig. 3). The 121 CNA drivers were classified across patients in different signaling pathways (Fig. 4). Gain of *ALK* copies known as a resistance mechanism to crizotinib was observed in 10/11 epithelial CTCs of P46. Multiple gains and losses in ALK-independent pathways were observed in all patient CTCs. Cell cycle-related and DNA repair-related pathways were the most prevalent with a total of 24 and 19 altered genes respectively. Multiple gains of *cyclin D3* (*CCND3*, chr6)—a temporal coordinator of the mitotic cycle—were found in 48.7% of CTC samples from crizotinib- and lorlatinib-resistant patients. Three crizotinib- (P46, P49, P50) and one lorlatinib- (P45) relapsing patient harbored gains of *septin 9* (*SEPT9*, chr17) in epithelial CTCs, a gene with a widely documented role in cytokinesis control^29^. Key kinases regulating cell cycle G1 progression were also altered in different CTC samples including *cyclin-dependent kinase 6* (*CDK6*, chr7) gain in CTC 1 of P43 and in single CTCs of P50, and *cyclin-dependent kinase 4* (*CDK4*, chr12) gains in 5/7 CTCs of P45 and the corresponding tumor biopsy. The most common CNA losses in cell cycle-related pathways included the tumor repressor *RB1* (33.3%) and adjacent cyclin-dependent kinase inhibitor genes (*CDKN2A* and *CDKN2B*, chr9) (30.8%). Gains of DNA damage-related proto-oncogenes *MDM2* (chr12) and *MDM4* (chr1) were frequent across CTC samples except in P43 and P46. Usually considered as an early event, the loss of tumor suppressor *TP53* was detected in 41% of CTC samples. However, despite multiple losses, at least one copy of the *TP53* gene was preserved in all evaluated samples. Complex alterations of chromosome 3 in CTC 6 of lorlatinib-relapsing P45 resulted in a complete loss (0 copy) of two homologous recombinations (HR)-related genes: *BRCA1 associated protein 1* (*BAP1*) and *Fanconi anemia complementation group D2* (*FANCD2*). In addition, the same CTC harbored a complete loss of mutL homolog 1 (MLH1), a DNA mismatch repair gene. The RTK/RAS-related pathway, which was previously found mutated^27^, was also altered with 15 CNAs. CTCs from P46, P49, and P45 (30.8% of CTC samples) harbored gains in *erb-b2 receptor tyrosine kinase 2* (*ERBB2*, chr17). Gains of *epidermal growth factor receptor* (*EGFR*, chr7), fibroblast growth factor receptor 1 (*FGFR1*, chr8), *v-ki-ras2 kirsten rat sarcoma 2 viral oncogene homolog* (*KRAS*, chr12), raf-1 proto-oncogene (*RAF1*, chr3), and alterations in downstream effectors of RTK/RAS signaling *mitogen-activated protein kinase kinase 4* (*MAP2K4*, chr17) and *mitogen-activated protein kinase kinase kinase kinase 1* (*MAP4K1*, chr19) were also detected. A total of six CNAs in PI3K-related pathways were detected, including *v-akt murine thymoma viral oncogene homolog 1* (*AKT1*, chr14) gains (33.3%). Gains of *c-myc* (*MYC*, chr8) proto-oncogene were observed in all patients and *MYCN* (chr2) was gained in various samples of P46, P49, P41, and P45. Drivers in other signaling pathways were also identified, notably genes involved in immune response that may be potentially implicated in adaptive immune evasion mechanisms of CTCs.

Although the interpretation of the data is limited by the small number of patients, we examined the CNA drivers that were exclusively present in crizotinib- or lorlatinib- resistant patients. The CNA drivers exclusively present in these groups were distributed in different pathways. However we observed that 16% (6/37) of the CNA drivers exclusively present in crizotinib-resistant were implicated in the immune response or cell cycle pathways, 14% (5/37) were implicated in DNA repair, and 11% (4/37) in cell adhesion. Forty percent (4/10) of the CNA drivers exclusively present in lorlatinib-resistant patients were involved in the cell cycle pathway. Overall, cell cycle and DNA repair were predominant among the 16 *ALK*-independent signaling pathways. Activation of RTK/RAS, PI3K, and MYC pathways resulting from gains may also be of clinical relevance. The variety of activated oncogenic drivers reflects a remarkable genomic heterogeneity of CTCs from patients at resistance to ALK-TKIs.

## Discussion

Although acquired *ALK* mutations remain an important resistance mechanism to ALK inhibitors currently guiding therapeutic decisions, heterogeneous ALK-independent signaling and non-mutation-driven pathways may equally influence cancer progression and treatment resistance. In the present work, we presented an exploratory comprehensive analysis of CNA heterogeneity in CTCs of six *ALK*-rearranged patients resistant to crizotinib or lorlatinib. To evaluate CTC heterogeneity, patients with ≥10 CTCs in 20 mL blood were selected allowing the characterization of 90 CTC samples according to their epithelial and/or *ALK*-positive phenotype. Phenotypic analysis revealed relatively homogeneous CTC phenotypes for each patient that were either ALK^+^ (*ALK*-rearranged)/CK^−^ or ALK^−^/CK^+^. CNA analysis performed on 43 CTC samples showed highly altered copy number profiles. Hierarchical clustering evidenced variable intra-patient CTC heterogeneity and wide inter-patient diversity, which was also observed in DNA content with a ploidy up to 6n. Finally, classification of CNA drivers in corresponding signaling pathways revealed the predominant activation of cell cycle and DNA repair pathways and that of RTK/RAS and PI3K to a smaller extent.

Using the CellSearch system, many studies have reported low counts of CTCs with epithelial characteristics in NSCLC, even at an advanced stage. We and others have shown that larger CTC numbers can be identified using various non EpCAM-based detection methods, most likely because CTCs that have lost their epithelial features and express epithelial-mesenchymal transition (EMT) markers can be missed by the CellSearch^30–32^. However, method robustness varied and the true tumor origin of these cells was questioned. Here, we demonstrate that (i) patients harbored predominantly either an ALK^+^/CK^−^ or an ALK^−^/CK^+^ CTC population, (ii) these two CTC populations displayed highly altered CNA profiles at resistance to ALK-TKIs. We previously reported that *ALK*-rearranged CTCs expressed a mesenchymal phenotype and high ALK protein expression in corresponding tumor tissue correlated with CK-negative areas, while tumor cells with lower CK harbored higher vimentin expression^19^. The present data confirm our previous results by showing that *ALK*-rearranged CTCs downregulate epithelial marker expression both at diagnosis and treatment resistance and therefore harbor an EMT phenotype that may contribute to escape ALK inhibition. Indeed EMT was found to be involved in cancer progression, metastasis, and drug resistance in different types of tumors, including NSCLC^33^. In addition, growing evidence suggests that EMT gene signature is predictive of resistance to TKI therapies in NSCLC^34,35^. Conversely, although the lack of ALK expression on epithelial CTCs may raise concerns that these cells may not be related to the patient primary lung cancer, our data suggest that the emergence of epithelial ALK-negative CTCs may drive ALK-TKIs resistance mechanisms through the activation of ALK-independent pathways.

Gain of *ALK* gene copies can result either from a true amplification—which occurs at a lower but variable frequency in NSCLC depending on the considered number of *ALK* copies—or from chromosome 2 polysomy—a frequent event in NSCLC, independent of *ALK*-rearrangement. Chromosome 2p gains have been identified through CGH assays in both squamous cell carcinoma (27.5%) and adenocarcinoma (20%)^36^. Although not dominant, *ALK* gene amplification has been reported as a resistance mechanism to crizotinib in vitro^37^ while an amplification of ALK gene has been reported in post-treatment tumor specimens of NSCLC patients^8,38^. We previously showed that an increase in the number of CTCs harboring a gain of *ALK* copies correlated with PFS in *ALK*-rearranged NSCLC patients treated with crizotinib^29^. Here, we identified a gain of *ALK* copies and chromosome 2 polysomy in 10/11 ALK^−^/CK^+^ CTCs of patient P46 who relapsed on crizotinib. This result is in agreement with studies revealing that amplification or gain of *ALK* gene is not involved in mRNA transcription and consequently in protein production^39^. We also observed that epithelial CTCs with a gain of *ALK* gene are heterogeneous in terms of number of *ALK* copies and cellular ploidy. However, almost all of these CTCs also harbored *MYCN* and *SEPT9* gains, which may act as potent oncogenic drivers to escape crizotinib therapy.

We previously reported heterogeneous CTC mutational profiles and detection of various mutations in bypass pathways from crizotinib and lorlatinib relapsing patients^27^. RTK/RAS was found to be the predominant mutated ALK-independent pathway revealed by CTCs. Here, 12% of CTCs acquired CNAs potentially implicated in RTK/RAS signaling pathway. Engagement of multiple proto-oncogenes including KRAS/HRAS as a bypass mechanism of resistance to ALK inhibitors has been reported by CNA analysis in tumor biopsies by McCoach et al.^9^, and, in concordance with our results, the authors found multiple gains in *EGFR*, *FGFR1,* and *NTRK1*. Activation of ERBB2 (HER2) has been reported as a potential off-target mechanism of resistance to crizotinib in *ALK*-rearranged tumors^40^. Similarly, our results showed the presence of *ERBB2* gains in 30.8% of CTCs from three patients indicating its potential importance in resistance to ALK inhibition. *MYC* gains and *CDKN2A/B* losses were found to be the most prevalent across all tumor types^11^. These alterations were the most frequent in CTC samples of patients resistant to ALK-TKIs in this study. This observation is also in agreement with previous data showing a high frequency of *MYC* alterations in lung cancer^41^. It is noteworthy that CNA driver classification revealed a predominant activation of cell cycle and DNA repair-related genes. CIN may lead to DNA repair-related gene amplification resulting in increased activation of DNA repair pathways. This may ensure high replicative capacities of CTCs and contribute to resistance to DNA-damaging anticancer therapies as observed for ERCC1 overexpression^42^. Similar to our study, alterations in cell cycle and DNA repair-related genes, like *ARID1A*, *BRCA1/2*, *TP53, CDKN2A/B, CDK4*/6, *CCND2* or *ATM* were also detected in tumor samples from ALK-positive NSCLC patients relapsing on second or third generations of TKI^10,43,44^. Inactivation of DNA damage response genes which increases genomic instability is now exploited via synthetic lethal approaches for NSCLC treatment^45^.

Therefore, CNAs in DNA repair-related genes frequently found in CTCs at resistance to ALK-TKIs further increase genomic instability and may consist in potent therapeutic targets.

CIN underpins much of the intratumor heterogeneity and promotes anticancer drug resistance, often leading to treatment failure and disease recurrence^46^^,47^. CIN was shown to be a predictor of poor outcome in NSCLC^13^ but it has not so far been specifically assessed in the different molecular subtypes of NSCLC. Here we deciphered a great heterogeneity of CNAs in CTCs harboring a high ploidy status (≥3n). This observation is consistent with previous reports showing higher tolerance to genomic alterations in aneuploid and polypoid cancer cells^13,46,48,49^. Lagging chromosomes and anaphasic bridges arising from under-replicated DNA may induce micronuclei formation or/and mitotic defects consequently leading to asymmetrical cell division and 3n ploidy. WGD is described as a one-off hit that generates aneuploidy and a key event during NSCLC evolution^13^. WGD was associated with poor prognosis in several cancer types^12,47^. Indeed, it consists of a transformative event during tumor evolution with a strong impact on the acquisition of deleterious genomic alterations^46,50^. Our report underlines a potential association between WGD and the accumulation of genomic CNAs in CTCs leading to increased tumor heterogeneity. Numerical CIN frequently arises from dysregulation of the cell cycle and cell division coordination. We showed that gains in *CCND3* and *SEPT9* were the most prevalent in CTCs of TKI-resistant *ALK*-positive patients, indicating the potential implication of cell cycle control in resistance mechanisms to ALK inhibitors.

Our study presents several limitations. First, we analyzed a limited number of patients at a single time-point. Patients with high CTC counts and possibly a more aggressive disease were selected. Both pre- and post-treatment samples are needed to demonstrate that a mechanism is acquired during treatment. Our samples do not allow monitoring a longitudinal change in the CNA profiles during treatment. Half the CTC samples passed the quality controls and were examined for their CNA content. This significant loss of CTC quality is a potential detrimental effect of therapies administered to the patients or a result of the various experimental processes undergone by CTCs. The tumor biopsy at resistance was obtained for P45 only. Despite these different limitations, our exploratory study presents original data on the phenotypical and genomic inter- and intra-patient diversity of CTCs at resistance to ALK-TKI. This may provide new hypotheses on metastatic progression and acquired resistance mechanisms that switch ALK dependence to alternate bypass pathways and open new perspectives for monitoring *ALK*-positive patients in this setting.

In conclusion, our exploratory study highlights the use of CNA profiling revealing CIN and WGD events in phenotypical subsets of CTCs as means to portray heterogeneous resistance mechanisms to ALK-TKIs. A real-time evaluation of single CTCs complementary to bulk sample analysis (i.e., tumor biopsies and ctDNA) in a larger cohort of patients is needed to confirm the clinical relevance of CTCs in depicting critical genomic events that drive resistance to ALK-TKIs in *ALK*-positive NSCLC.

## Methods

### Patients

The study (IDRCB2008-A00585-50) was conducted at Gustave Roussy (Villejuif, France) in accordance with the Declaration of Helsinki. It was authorized by the French national regulation agency ANSM (*Agence Nationale de Sécurité du Medicament et des produits de santé*) and approved by the Ethics Committee and our institutional review board. Informed written consent was obtained from all patients. Stage IV *ALK*-rearranged NSCLC patients were recruited into the study. *ALK*-rearrangement was tested in tumor by FISH, immunochemistry or reverse transcription-polymerase chain reaction. Peripheral blood samples were collected into EDTA tubes for germline DNA samples, Transfix/EDTA tubes for CTC detection and isolation by CellSearch and DEPArray, and CellSave tubes for CTC isolation by CellSorting at progression disease (PD). Blood samples were performed on treatment or within 1 week of treatment discontinuation. Medical records were reviewed, and clinical characteristics were collected retrospectively.

### Description of the strategies for CTC enrichment, detection, and isolation

#### Enrichment and detection of CTCs by the CellSearch and isolation of CTC-enriched using the DEPArray system

CTC enrichment using the CellSearch system (Menarini Silicon Biosystems) was performed according to the manufacturer’s protocol using the CTC kit on 7.5 mL of blood collected in CellSave tubes as previously described^51^. At the end of the run, the cartridge was immediately removed from the MagNest device to prevent cells from sticking to the cartridge surface and stored at 4 °C in the dark. CTCs were identified as DAPI^+^/CK^+^/CD45^−^ cells and enumerated according to the manufacturer’s guidelines. Cells were removed from the CellSearch cartridge and CTCs were isolated using the DEPArray system (Menarini Silicon Biosystems) according to the manufacturer’s protocol.

#### Enrichment of CTCs by RosetteSep, immunofluorescent staining and isolation of single CTCs by fluorescence-activated cell sorter (FACS)

Negative selection of CTCs by the RosetteSep Human CD45 Depletion Cocktail (StemCell Technologies, https://www.stemcell.com/technical-resources/product-information/product-information-sheet.html) was performed according to the manufacturer’s protocol starting from 20 mL blood samples collected in CellSave tubes. Cells collected were washed with PBS 1X and centrifuged 5 min at 1600 rpm. The cell pellet was suspended with 100 µL of fixative solution medium A from the Fix&Perm kit (Thermo Fisher Scientific) and washed with PBS 1×. Cells were centrifuged 5 min at 1300 rpm. The cell pellet was re-suspended with 100 µL of permeabilization solution medium B from the Fix&Perm kit and a rabbit anti-ALK (1/400, Cell Signaling, D5F3, #3633) monoclonal antibody and incubated 20 min at room temperature (RT). After a PBS1× wash and a centrifugation, a secondary goat anti-rabbit AF488 antibody (1/800, Life Technologies, #A11070) and 50 µL of staining reagent containing cytokeratins (CK)-PE (CK 8, 18, 19) and CD45-APC antibodies from the CellSearch reagent kit (1/6, Menarini Silicon Biosystem, #7900001) was added. The cell suspension was incubated 20 min in the dark at RT and then centrifuged. The cell pellet was re-suspended in 300 µL of PBS 1X and kept at +4 °C. Hoechst 33342 (16.7 µg/mL, Merck, #B2261) was added before cell sorting. Individual CTC isolation was performed using a BD FACS ARIA III cell sorter (BD Biosciences) equipped with four lasers (a 405 nm laser, a 488 nm laser, a 561 nm laser, and a 640 nm laser). The system was run at 20 psi pressure, a 100 µm nozzle and the yield precision mode. Cell sorting started by gating Hoechst-positive elements. The second gate enabled selecting CD45-APC-negative events. Three populations including Hoechst^+^/CD45-APC^−^/CK-PE^+^/ALK-AF488^−^, Hoechst^+^/CD45-APC^−^/CK-PE^−^/ALK-AF488^+^ and Hoechst^+^/CD45-APC^−^/CK-PE^+^/ALK-AF488^+^ cells were sorted and recovered in 96-well plate. As a control, 200 Hoechst^+^/CD45-APC^+^/CK-PE^−^/ALK-AF488^−^ cells were sorted in a well. Plates were centrifuged 10 min at 1200 rpm and frozen at −20 °C for at least 30 min. At the end of the study, FACS data were re-analyzed so as to have a homogeneous gating for all patients. Cells initially considered as putative CTCs and sequenced were discarded after refining the gating.

#### Whole-genome amplification and quality controls

Whole-genome amplification (WGA) was performed using the Ampli1 WGA kit (Menarini Silicon Biosystems) according to the manufacturer’s instructions. The quality of Ampli1 WGA products was checked to assess Genome Integrity Index as previously reported^52^. Briefly, for the multiplex PCR (QC2 assay), 0.30 µL (per reaction) of WGA product was used in 5 µL of Dream Taq Green PCR Master Mix (2X) (ThermoFisher Scientific, # K1081), 1 µL of Primer Mix QC2 (10X) (Eurofins Genomics) and 3.70 µL of water nuclease-free. PCR was started with a first step at 95 °C for 4 min, followed by 35 cycles of 95 °C for 30 s, 58 °C for 30 s and 72 °C for 90 s, and a final elongation step of 7 min at 72 °C. To determine the genome integrity index, PCR products were visualized on a 2% agarose gel.

#### Library preparation and low-pass whole-genome sequencing

This workflow was done by Menarini Silicon Biosystems. Ampli1 LowPass kit for Illumina (Menarini Silicon Biosystems) was used for preparing low-pass whole-genome sequencing (LP-WGS) libraries from single cells. For high-throughput processing, the manufacturer’s procedure was implemented in a fully automated workflow on a STARlet Liquid Handling Robot (Hamilton). Ampli1 LowPass libraries were normalized and sequenced by HiSeq 2500 instrument using 150 SR rapid-run mode.

#### Bioinformatic workflow for low-pass whole-genome sequencing

For the LP-WGS, the workflow of the bioinformatics analysis was done by Menarini Silicon Biosystems.

#### Sequence alignment

The obtained FASTQ files were aligned to the hg19 human reference sequence using Burrows-Wheeler Aligner version 0.7.12 (BWA) using mem algorithm with default parameters^53^.

#### CNA calling and ploidy determination

CNAs in the data were identified using Control-FREEC software (version 11.0) with control-free mode and coefficient of variation parameter set to 0.06^54^. Ploidy level was automatically estimated by Menarini Silicon Biosystems pipeline for each library based on best fitting of profiles to underlying copy number levels with different ploidies ranging from 2 to 6. For each ploidy the root mean square error (RMSE) and percentage of genome explained are calculated using Control-FREEC. Ploidy is selected based on minimization of RMSE and maximization of the percentage of genome explained. All the other Control-FREEC parameters were set to default values.

#### Hierarchical clustering

For clustering analysis, starting from the median ratio obtained by the Control-FREEC software, each profile was normalized on fixed bins length (weighted mean on 250,000 bp windows) and log2 values were calculated. Hierarchical clustering was performed using the “euclidean” distance metric and “ward” clustering method. The heatmap shows the log2 values between −2 and +2 with positive values in red (gain) and negative values in blue (loss). Copy-number profile plots and clustering heatmaps were automatically generated by Menarini Silicon Biosystems pipeline.

### Reporting summary

Further information on research design is available in the Nature Research Reporting Summary linked to this article.

## Supplementary information

Supplementary Information

REPORTING SUMMARY

Supplementary Data

## Data Availability

The LowPass whole-genome sequencing data are available in a public repository from the https://ega-archive.org/ website under the accession code EGAS00001005301. All the other data supporting the findings of this study are available within the article and its Supplementary Information Files.
