# Supplementary material for: Circulating tumor cell copy-number heterogeneity in ALK-rearranged non-small-cell lung cancer resistant to ALK inhibitors
Source: NPJ Precis Oncol. 2021 Jul 16;5:67. doi: 10.1038/s41698-021-00203-1 (PMC8285416; doi:10.1038/s41698-021-00203-1)
Supplement: Supplementary file 1 — Supplementary Information [file 41698_2021_203_MOESM1_ESM.pdf]

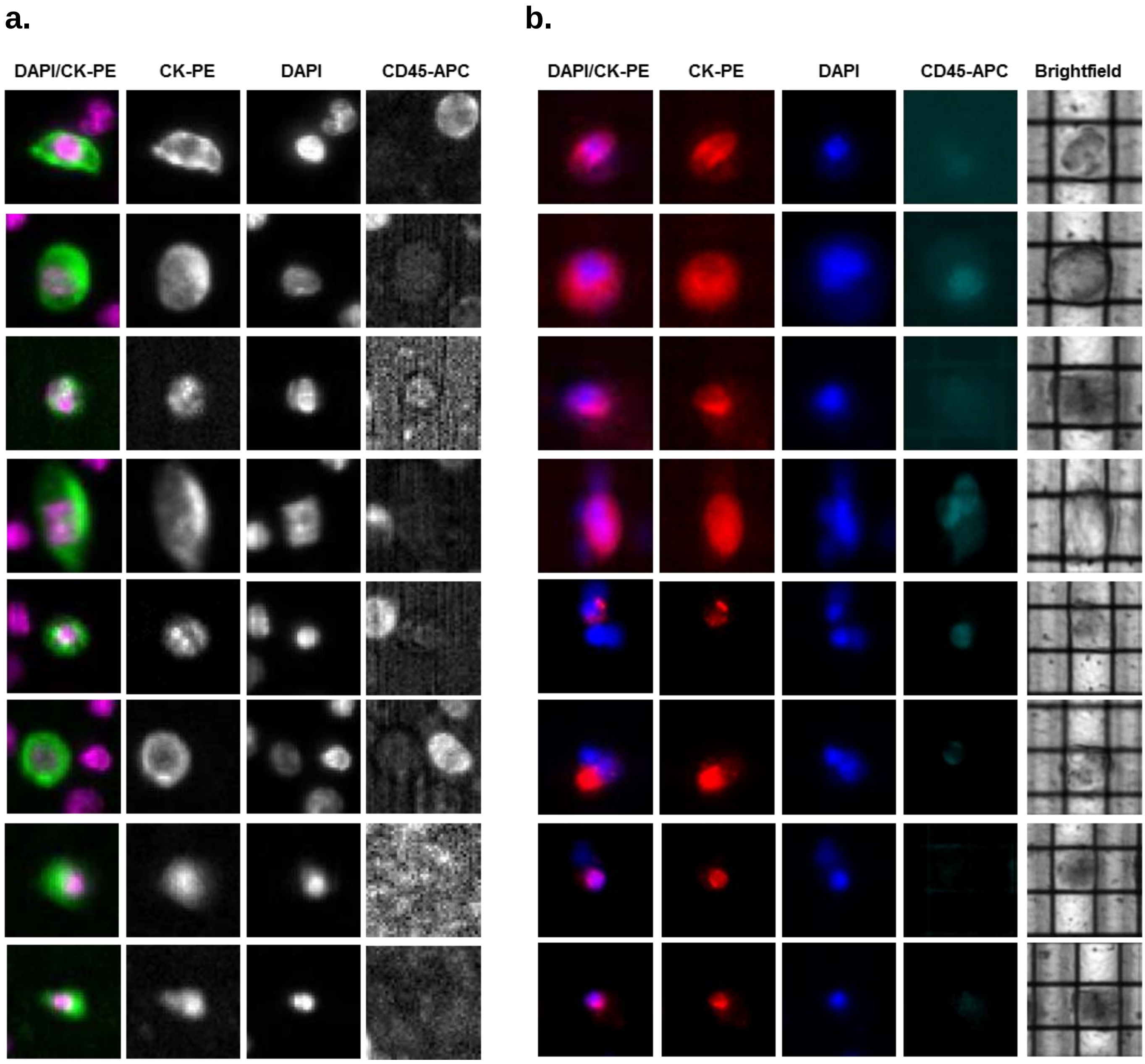


**Supplementary Figure 1.** Isolated CTCs by DEPArray. (**a**) Enrichment and detection of CTCs by the CellSearch. (**b**) Isolation of single CTCs using the DEPArray. The *VHL*$^{R161}$ mutation was previously identified in one P49 CK$^{+}$ CTC (27).

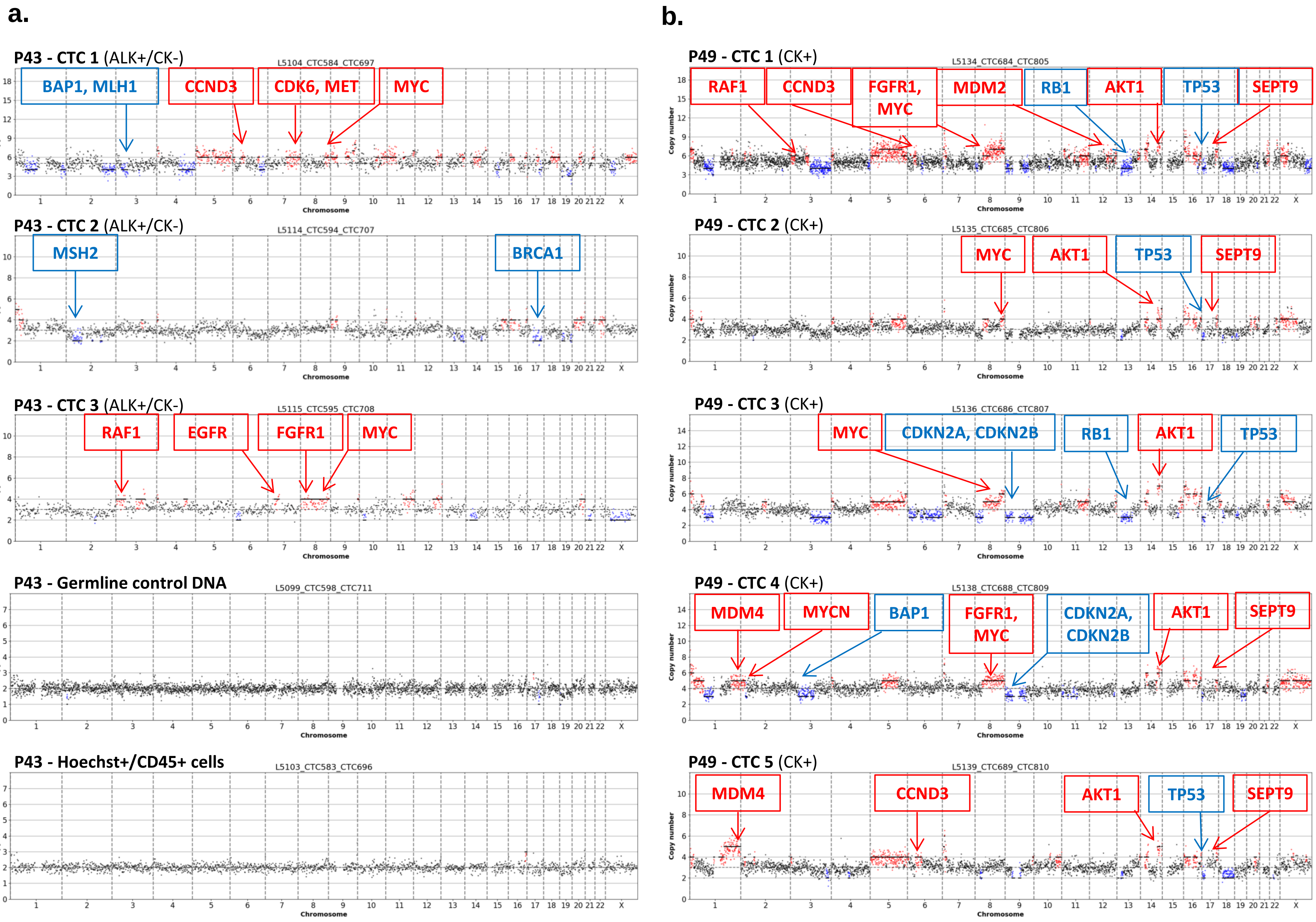
a.
b.
P43 - CTC 1 (ALK+/CK-)
L5104_CTC584_CTC697
BAP1, MLH1
CCND3
CDK6, MET
MYC
P43 - CTC 2 (ALK+/CK-)
L5114_CTC594_CTC707
MSH2
BRCA1
P43 - CTC 3 (ALK+/CK-)
L5115_CTC595_CTC708
RAF1
EGFR
FGFR1
MYC
P43 - Germline control DNA
L5099_CTC598_CTC711
P43 - Hoechst+/CD45+ cells
L5103_CTC583_CTC696
P49 - CTC 1 (CK+)
L5134_CTC684_CTC805
RAF1
CCND3
FGFR1, MYC
MDM2
RB1
AKT1
TP53
SEPT9
P49 - CTC 2 (CK+)
L5135_CTC685_CTC806
MYC
AKT1
TP53
SEPT9
P49 - CTC 3 (CK+)
L5136_CTC686_CTC807
MYC
CDKN2A, CDKN2B
RB1
AKT1
TP53
P49 - CTC 4 (CK+)
L5138_CTC688_CTC809
MDM4
MYCN
BAP1
FGFR1, MYC
CDKN2A, CDKN2B
AKT1
SEPT9
P49 - CTC 5 (CK+)
L5139_CTC689_CTC810
MDM4
CCND3
AKT1
TP53
SEPT9
Copy number
Chromosome

b.

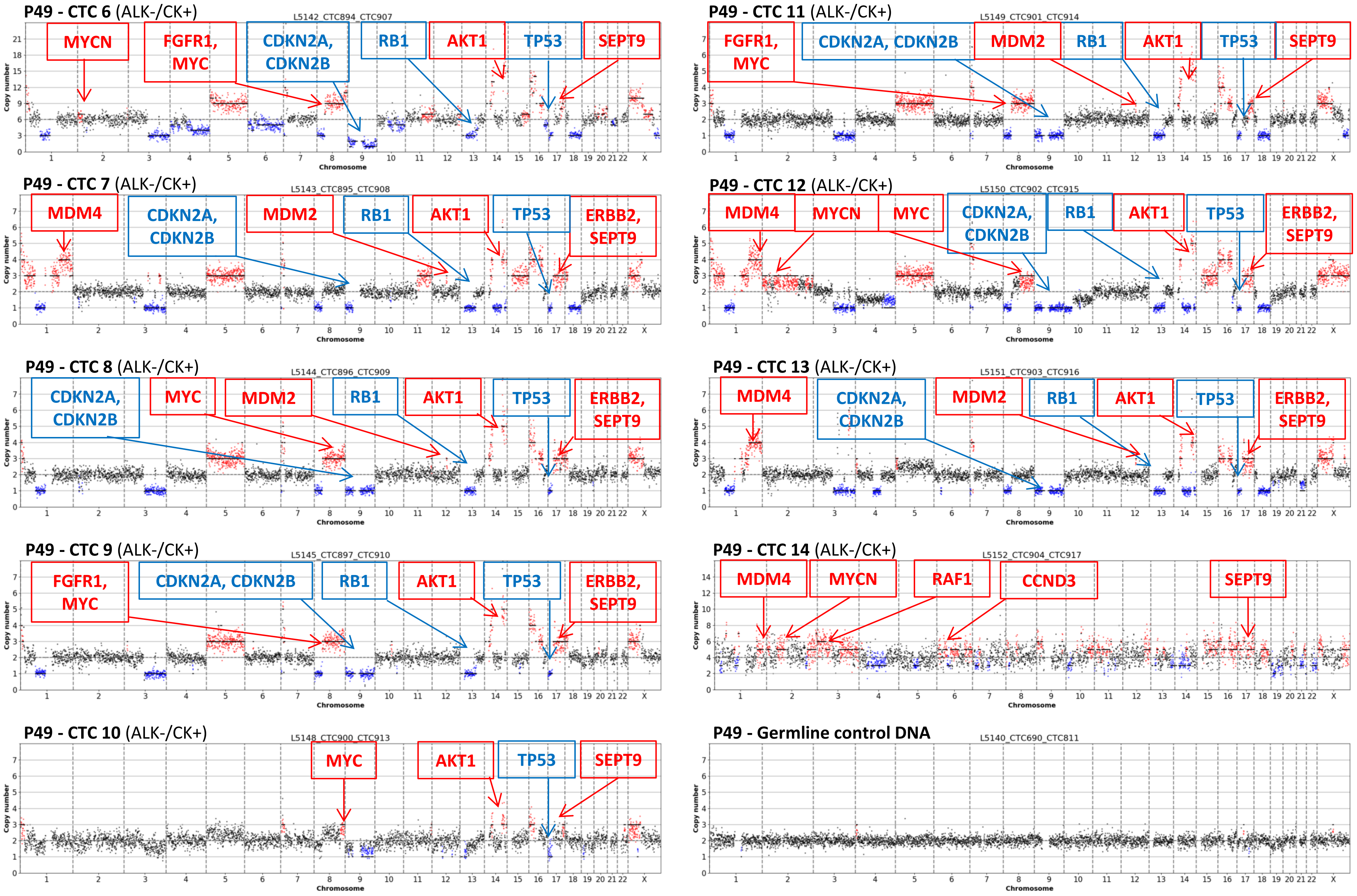
P49 - CTC 6 (ALK-/CK+)
L5142_CTC894_CTC907
MYCN
FGFR1, MYC
CDKN2A, CDKN2B
RB1
AKT1
TP53
SEPT9
P49 - CTC 11 (ALK-/CK+)
L5149_CTC901_CTC914
FGFR1, MYC
CDKN2A, CDKN2B
MDM2
RB1
AKT1
TP53
SEPT9
P49 - CTC 7 (ALK-/CK+)
L5143_CTC895_CTC908
MDM4
CDKN2A, CDKN2B
MDM2
RB1
AKT1
TP53
ERBB2, SEPT9
P49 - CTC 12 (ALK-/CK+)
L5150_CTC902_CTC915
MDM4
MYCN
MYC
CDKN2A, CDKN2B
RB1
AKT1
TP53
ERBB2, SEPT9
P49 - CTC 8 (ALK-/CK+)
L5144_CTC896_CTC909
CDKN2A, CDKN2B
MYC
MDM2
RB1
AKT1
TP53
ERBB2, SEPT9
P49 - CTC 13 (ALK-/CK+)
L5151_CTC903_CTC916
MDM4
CDKN2A, CDKN2B
MDM2
RB1
AKT1
TP53
ERBB2, SEPT9
P49 - CTC 9 (ALK-/CK+)
L5145_CTC897_CTC910
FGFR1, MYC
CDKN2A, CDKN2B
RB1
AKT1
TP53
ERBB2, SEPT9
P49 - CTC 14 (ALK-/CK+)
L5152_CTC904_CTC917
MDM4
MYCN
RAF1
CCND3
SEPT9
P49 - CTC 10 (ALK-/CK+)
L5148_CTC900_CTC913
MYC
AKT1
TP53
SEPT9
P49 - Germline control DNA
L5140_CTC690_CTC811
Copy number
Chromosome
1 2 3 4 5 6 7 8 9 10 11 12 13 14 15 16 17 18 19 20 21 22 X

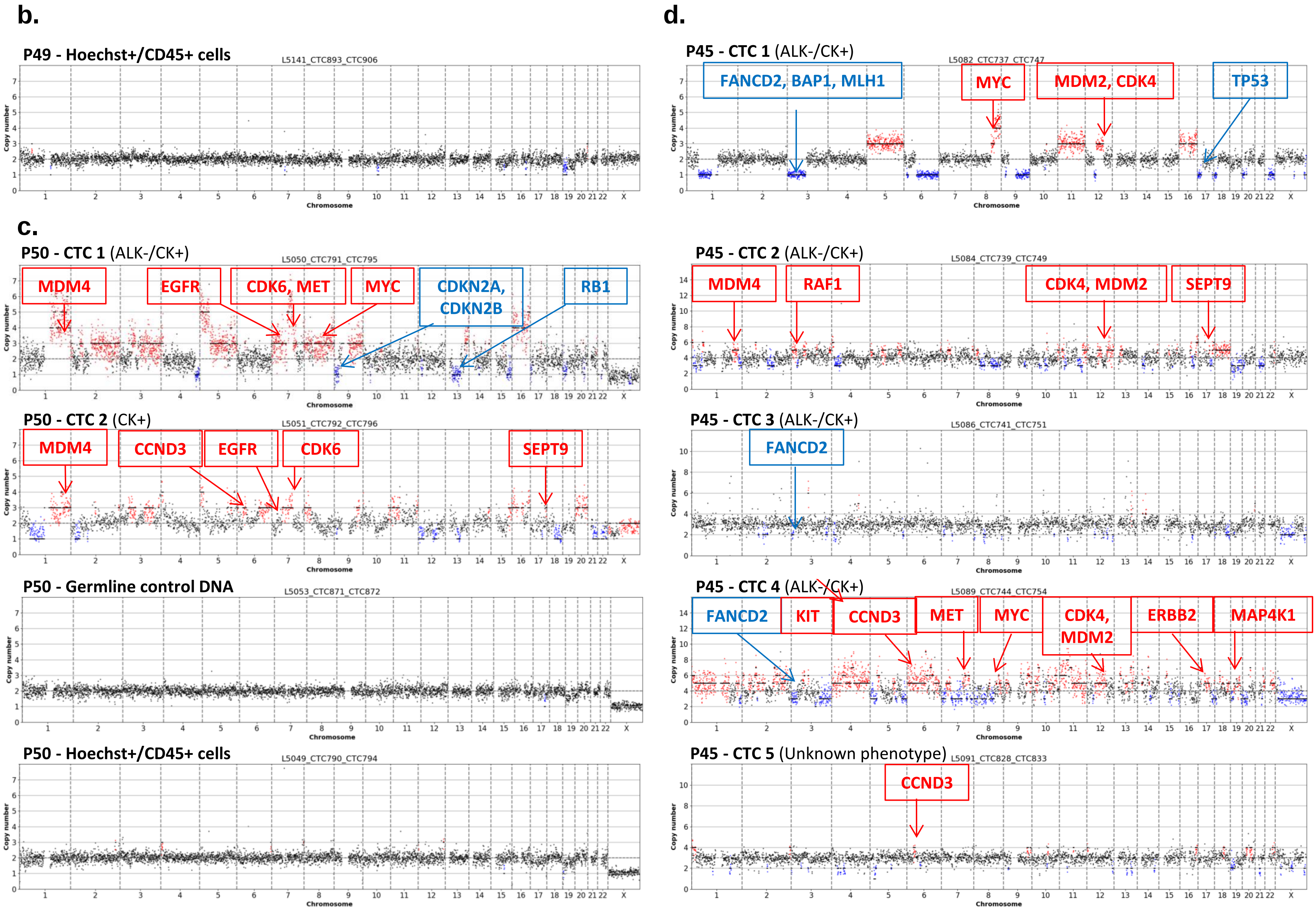
b.
P49 - Hoechst+/CD45+ cells
L5141_CTC893_CTC906
c.
P50 - CTC 1 (ALK-/CK+)
L5050_CTC791_CTC795
MDM4
EGFR
CDK6, MET
MYC
CDKN2A, CDKN2B
RB1
P50 - CTC 2 (CK+)
L5051_CTC792_CTC796
MDM4
CCND3
EGFR
CDK6
SEPT9
P50 - Germline control DNA
L5053_CTC871_CTC872
P50 - Hoechst+/CD45+ cells
L5049_CTC790_CTC794
d.
P45 - CTC 1 (ALK-/CK+)
L5082_CTC737_CTC747
FANCD2, BAP1, MLH1
MYC
MDM2, CDK4
TP53
P45 - CTC 2 (ALK-/CK+)
L5084_CTC739_CTC749
MDM4
RAF1
CDK4, MDM2
SEPT9
P45 - CTC 3 (ALK-/CK+)
L5086_CTC741_CTC751
FANCD2
P45 - CTC 4 (ALK-/CK+)
L5089_CTC744_CTC754
FANCD2
KIT
CCND3
MET
MYC
CDK4, MDM2
ERBB2
MAP4K1
P45 - CTC 5 (Unknown phenotype)
L5091_CTC828_CTC833
CCND3
Copy number
Chromosome

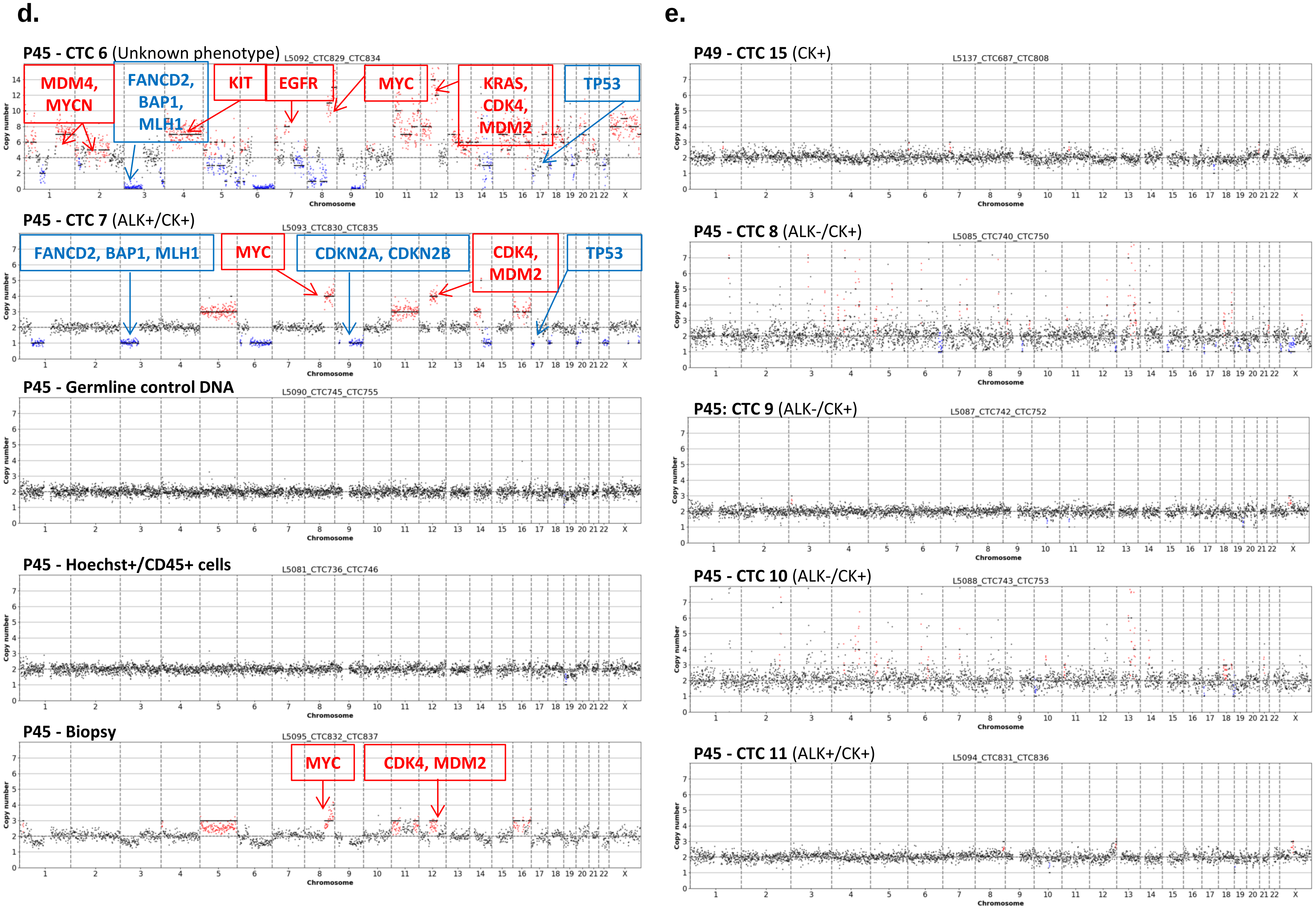

d.
P45 - CTC 6 (Unknown phenotype)
L5092_CTC829_CTC834
MDM4, MYCN
FANCD2, BAP1, MLH1
KIT
EGFR
MYC
KRAS, CDK4, MDM2
TP53
P45 - CTC 7 (ALK+/CK+)
L5093_CTC830_CTC835
FANCD2, BAP1, MLH1
MYC
CDKN2A, CDKN2B
CDK4, MDM2
TP53
P45 - Germline control DNA
L5090_CTC745_CTC755
P45 - Hoechst+/CD45+ cells
L5081_CTC736_CTC746
P45 - Biopsy
L5095_CTC832_CTC837
MYC
CDK4, MDM2
e.
P49 - CTC 15 (CK+)
L5137_CTC687_CTC808
P45 - CTC 8 (ALK-/CK+)
L5085_CTC740_CTC750
P45: CTC 9 (ALK-/CK+)
L5087_CTC742_CTC752
P45 - CTC 10 (ALK-/CK+)
L5088_CTC743_CTC753
P45 - CTC 11 (ALK+/CK+)
L5094_CTC831_CTC836
Copy number
Chromosome

**Supplementary Figure 2.** Low-pass whole-genome CNA profiles of CTC samples from *ALK*-positive patients at resistance to ALK-TKIs. CNAs in predominant pathways are annotated (gain in red, loss in blue). (**a**) CNA profiles of CTCs, corresponding leucocytes and germline DNA from patient P43 resistant to crizotinib. (**b**) CNA profiles of CTCs, corresponding leucocytes and germline DNA from patient P49 resistant to crizotinib. (**c**) CNA profiles of CTCs, corresponding leucocytes and germline DNA from patient P50 resistant to crizotinib. (**d**) CNA profiles of CTCs, corresponding tumor biopsy, leucocytes and germline DNA from patient P45 resistant to lorlatinib. (**e**) Flat CNA profiles of $CK^{+}$ cells from P49 and P45.

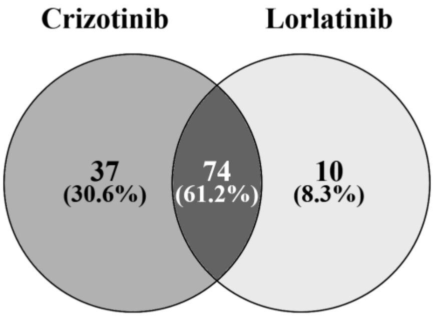


**Supplementary Figure 3.** Numbers and percentages of CNA drivers identified in crizotinib-resistant patients only, in lorlatinib-resistant patients only and in both.

**Supplementary Table 1.** Detailed list of CNAs identified in tumor samples (38 CTC samples and one tumor biopsy) of *ALK*-positive patients at resistance to ALK-TKIs.

| ALK TKI | Patient | Sample ID | WGA QC GII ( /4) | LowPass Genome QC | | | |
|---|---|---|---|---|---|---|---|
| | | | | Read counts | DLRS | LMAD | R50 |
| Crizotinib | P43 | CTC 1 | 3 | 718,457 | 0,25 | 0,23 | 45 |
| Crizotinib | P43 | CTC 2 | 3 | 638,892 | 0,24 | 0,23 | 45 |
| Crizotinib | P43 | CTC 3 | 3 | 399,283 | 0,24 | 0,23 | 45 |
| Crizotinib | P46 | CTC 1 | 4 | 771,976 | 0,25 | 0,25 | 46 |
| Crizotinib | P46 | CTC 2 | 4 | 851,956 | 0,28 | 0,28 | 46 |
| Crizotinib | P46 | CTC 3 | 4 | 855,924 | 0,24 | 0,24 | 46 |
| Crizotinib | P46 | CTC 4 | 4 | 876,131 | 0,22 | 0,21 | 46 |
| Crizotinib | P46 | CTC 5 | 4 | 838,436 | 0,25 | 0,24 | 46 |
| Crizotinib | P46 | CTC 6 | 3 | 1,030,049 | 0,28 | 0,30 | 46 |
| Crizotinib | P46 | CTC 7 | 3 | 1,122,952 | 0,31 | 0,32 | 46 |
| Crizotinib | P46 | CTC 8 | 4 | 737,347 | 0,24 | 0,26 | 46 |
| Crizotinib | P46 | CTC 9 | 4 | 835,369 | 0,26 | 0,26 | 45 |
| Crizotinib | P46 | CTC 10 | 4 | 957,580 | 0,27 | 0,26 | 45 |
| Crizotinib | P46 | CTC 11 | 4 | 1,026,747 | 0,32 | 0,33 | 46 |
| Crizotinib | P49 | CTC 1 | 4 | 1,696,671 | 0,23 | 0,64 | 46 |
| Crizotinib | P49 | CTC 2 | 4 | 1,029,086 | 0,22 | 0,22 | 45 |
| Crizotinib | P49 | CTC 3 | 3 | 868,550 | 0,23 | 0,67 | 45 |
| Crizotinib | P49 | CTC 4 | 4 | 1,125,220 | 0,22 | 0,42 | 46 |
| Crizotinib | P49 | CTC 5 | 4 | 1,050,782 | 0,22 | 0,22 | 45 |
| Crizotinib | P49 | CTC 6 | 3 | 989,475 | 0,27 | 0,64 | 44 |
| Crizotinib | P49 | CTC 7 | 3 | 1,452,899 | 0,25 | 0,22 | 45 |
| Crizotinib | P49 | CTC 8 | 3 | 1,445,407 | 0,23 | 0,21 | 44 |
| Crizotinib | P49 | CTC 9 | 4 | 1,319,753 | 0,24 | 0,20 | 46 |
| Crizotinib | P49 | CTC 10 | 4 | 1,411,964 | 0,23 | 0,59 | 46 |
| Crizotinib | P49 | CTC 11 | 3 | 1,579,673 | 0,24 | 0,20 | 44 |
| Crizotinib | P49 | CTC 12 | 4 | 1,661,213 | 0,26 | 0,42 | 47 |
| Crizotinib | P49 | CTC 13 | 4 | 1,600,028 | 0,25 | 0,41 | 46 |
| Crizotinib | P49 | CTC 14 | 3 | 1,390,551 | 0,29 | 0,91 | 46 |
| Crizotinib | P49 | CTC 15 | 3 | 1,092,214 | 0,22 | 0,21 | 46 |
| Crizotinib | P50 | CTC 1 | 3 | 1,675,152 | 0,31 | 0,32 | 45 |
| Crizotinib | P50 | CTC 2 | 2* | 934,641 | 0,27 | 0,55 | 44 |
| Lorlatinib | P41 | CTC 1 | 1* | 1,279,226 | 0,33 | 0,38 | 46 |
| Lorlatinib | P45 | CTC 1 | 4 | 1,363,737 | 0,23 | 0,20 | 46 |
| Lorlatinib | P45 | CTC 2 | 4 | 1,207,893 | 0,24 | 0,24 | 46 |
| Lorlatinib | P45 | CTC 3 | 4 | 1,108,999 | 0,27 | 0,25 | 47 |
| Lorlatinib | P45 | CTC 4 | 4 | 1,137,825 | 0,29 | 0,78 | 46 |
| Lorlatinib | P45 | CTC 5 | 3 | 1,097,406 | 0,23 | 0,21 | 47 |
| Lorlatinib | P45 | CTC 6 | 3 | 805,981 | 0,51 | 0,71 | 45 |
| Lorlatinib | P45 | CTC 7 | 2* | 782,591 | 0,23 | 0,19 | 47 |
| Lorlatinib | P45 | CTC 8 | 4 | 1,239,079 | 0,31 | 0,44 | 48 |
| Lorlatinib | P45 | CTC 9 | 4 | 1,139,258 | 0,21 | 0,19 | 46 |
| Lorlatinib | P45 | CTC 10 | 4 | 1,027,209 | 0,31 | 0,41 | 47 |
| Lorlatinib | P45 | CTC 11 | 4 | 948,745 | 0,22 | 0,19 | 47 |
| Lorlatinib | P45 | Biopsy | 4 | 656,066 | 0,21 | 0,41 | 46 |

Abbreviations: DLRS, derivative log ratio spread is a measure of point-to-point consistency or noisiness in copy number along the genome (expected = 0,2-0,3); LMAD, absolute deviation from local median copy number (expected = 0,2-0,3); R50, percentage of target (total bp of genome covered) covered by 50% of reads. Low values (<20%) indicate low WGA/library complexity.
DLRS and LMAD are calculated with a subsampled number of 200,000 reads.
* The three CTC samples with lower Genome Integrity Index (GII) passed sequencing QC and 43 CTC samples in total were analyzed by the LowPass WGS.

**Supplementary Table 2**. Whole Genome Amplification and LowPass Genome quality controls
