# Supplementary material for: Circulating tumor cell copy-number heterogeneity in ALK-rearranged non-small-cell lung cancer resistant to ALK inhibitors
Source: NPJ Precis Oncol. 2021 Jul 16;5:67. doi: 10.1038/s41698-021-00203-1 (PMC8285416; doi:10.1038/s41698-021-00203-1)
Supplement: Supplementary file 2 — REPORTING SUMMARY [file 41698_2021_203_MOESM2_ESM.pdf]

nature research

Corresponding author(s): Françoise Farace

Last updated by author(s): Mar 25, 2021

Reporting Summary

Nature Research wishes to improve the reproducibility of the work that we publish. This form provides structure for consistency and transparency in reporting. For further information on Nature Research policies, see our Editorial Policies and the Editorial Policy Checklist.

Statistics

For all statistical analyses, confirm that the following items are present in the figure legend, table legend, main text, or Methods section.

n/a	Confirmed	
☒	☐	The exact sample size (*n*) for each experimental group/condition, given as a discrete number and unit of measurement
☒	☐	A statement on whether measurements were taken from distinct samples or whether the same sample was measured repeatedly
☒	☐	The statistical test(s) used AND whether they are one- or two-sided<br>*Only common tests should be described solely by name; describe more complex techniques in the Methods section.*
☒	☐	A description of all covariates tested
☒	☐	A description of any assumptions or corrections, such as tests of normality and adjustment for multiple comparisons
☒	☐	A full description of the statistical parameters including central tendency (e.g. means) or other basic estimates (e.g. regression coefficient) AND variation (e.g. standard deviation) or associated estimates of uncertainty (e.g. confidence intervals)
☒	☐	For null hypothesis testing, the test statistic (e.g. *F*, *t*, *r*) with confidence intervals, effect sizes, degrees of freedom and *P* value noted<br>*Give P values as exact values whenever suitable.*
☒	☐	For Bayesian analysis, information on the choice of priors and Markov chain Monte Carlo settings
☒	☐	For hierarchical and complex designs, identification of the appropriate level for tests and full reporting of outcomes
☒	☐	Estimates of effect sizes (e.g. Cohen's *d*, Pearson's *r*), indicating how they were calculated

Our web collection on statistics for biologists contains articles on many of the points above.

Software and code

Policy information about availability of computer code

Data collection	Collection of FACS data was performed using the BD FACS Diva software v6.1.3. Collection of CellSearch data was performed using the Celltracks analyzer II.
Data analysis	- FACS data were analyzed using the Kaluza software (1.5a, Beckman Coulter). CTC analysis by Cellsearch was performed using Celltracks analyzer II.<br>- The bioinformatic analysis of the Lowpass-whole genome sequencing workflow was done by Menarini Silicon Biosystems.<br>The obtained FASTQ files were aligned to the hg19 human reference sequence using Burrows-Wheeler Aligner version 0.7.12 (BWA) using mem algorithm with default parameters. Copy number alterations were identified using Control-FREEC software (version 11.0) with control-free mode and coefficient of variation parameter set to 0.06. For each ploidy the root mean square error (RMSE) and percentage of genome explained are calculated using Control-FREEC. Ploidy is selected based on minimization of RMSE and maximization of percentage of genome explained. All the other Control-FREEC parameters were set to default values.<br>For clustering analysis, starting from the median ratio obtained by the Control-FREEC software, each profile was normalized on fixed bins length (weighted mean on 250.000 bp windows) and log2 values were calculated. Hierarchical clustering was performed using the “euclidean” distance metric and “ward” clustering method.

For manuscripts utilizing custom algorithms or software that are central to the research but not yet described in published literature, software must be made available to editors and reviewers. We strongly encourage code deposition in a community repository (e.g. GitHub). See the Nature Research guidelines for submitting code & software for further information.

Data

Policy information about availability of data

All manuscripts must include a data availability statement. This statement should provide the following information, where applicable:

- Accession codes, unique identifiers, or web links for publicly available datasets
- A list of figures that have associated raw data
- A description of any restrictions on data availability

All data supporting the findings of this study are available within the article and its supplementary information files and from the corresponding author (FF) upon request.

Field-specific reporting

Please select the one below that is the best fit for your research. If you are not sure, read the appropriate sections before making your selection.

☒ Life sciences ☐ Behavioural & social sciences ☐ Ecological, evolutionary & environmental sciences

For a reference copy of the document with all sections, see nature.com/documents/nr-reporting-summary-flat.pdf

Life sciences study design

All studies must disclose on these points even when the disclosure is negative.

Sample size	Six patients who had given an informed consent for blood sampling were recruited. No statistical methods were used to predetermine sample size. This cohort of patients (n=6) was sufficient for the exploratory analysis performed in this study.
Data exclusions	For the copy number alterations analysis, several filters were applied in order to generate high-confidence alterations from CTCs. The filters applied are indicated in the Methods. They were established on the basis of previous publications.
Replication	Analysis of unique patient samples at resistance to treatment. Can not be replicated.
Randomization	There is no experiments requiring randomization since the study focused on the analysis of a single time point (resistance to ALK inhibitor).
Blinding	Blinding was not possible

Reporting for specific materials, systems and methods

We require information from authors about some types of materials, experimental systems and methods used in many studies. Here, indicate whether each material, system or method listed is relevant to your study. If you are not sure if a list item applies to your research, read the appropriate section before selecting a response.

Materials & experimental systems

n/a	Involved in the study	
☐	☒	Antibodies
☒	☐	Eukaryotic cell lines
☒	☐	Palaeontology and archaeology
☒	☐	Animals and other organisms
☐	☒	Human research participants
☐	☒	Clinical data
☒	☐	Dual use research of concern

Methods

n/a	Involved in the study	
☒	☐	ChIP-seq
☐	☒	Flow cytometry
☒	☐	MRI-based neuroimaging

Antibodies

Antibodies used	anti-Cytokeratins (CK)-PE (CK 8, 18, 19) and anti-CD45-APC antibodies from the CellSearch reagent kit (7900001, Menarini Silicon Biosystem<br>anti-ALK rabbit monoclonal antibody (D5F3) from Cell Signaling (3633S)
Validation	https://www.cellsearchctc.com/product-systems-overview/cellsearch-ctc-kit<br>https://www.cellsignal.com/products/primary-antibodies/alk-d5f3-xp-rabbit-mab/3633

Human research participants

Policy information about studies involving human research participants

Population characteristics	Six ALK-rearranged non-small cell lung cancer patients were sampled at resistance to ALK-inhibitors (crizotinib, lorlatinib). All the patient characteristics relevant for the study are presented in the Table 1 of the manuscript.<br>Inclusion criteria:<br>- Patients with solid metastatic tumor.<br>- Patients with > 18 years of age.<br>- Written informed consent.<br>Exclusion criteria:<br>- Patients protected by law, in accordance with articles L1121-5 to L1121-8 of the Public Health Code.<br>- Mental pathology which could interfere with the smooth running of the study.<br>- Not consenting.
Recruitment	Patients were recruited in the study IDRCB2008-A00585-50 was conducted at Gustave Roussy (Villejuif, France) in accordance with the Declaration of Helsinki.
Ethics oversight	The study was authorized by the French national regulation agency ANSM (Agence Nationale de Sécurité du Medicament et des produits de santé) and approved by the Ethics Committee and our institutional review board. Informed written consent was obtained from all patients

Note that full information on the approval of the study protocol must also be provided in the manuscript.

Clinical data

Policy information about clinical studies

All manuscripts should comply with the ICMJE guidelines for publication of clinical research and a completed CONSORT checklist must be included with all submissions.

Clinical trial registration	IDRCB2008-A00585-50
Study protocol	The IDRCB2008-A00585-50 full trial protocol can be accessed at the Department of Clinical Research, Institut Gustave Roussy (Villejuif, France).
Data collection	Patients were treated at Institut Gustave Roussy (IGR). Patient follow up and data (e. g. clinical characteristics, treatments) collection were performed at IGR.
Outcomes	The study was focused on the analysis of copy number alterations occuring in circulating tumor cells at resistace to ALK inhibitor treatment. In one patient, copy number alterations were in parrallel analysed in the tumor biopsy sampled at resistance.

Flow Cytometry

Plots

Confirm that:

☒ The axis labels state the marker and fluorochrome used (e.g. CD4-FITC).

☒ The axis scales are clearly visible. Include numbers along axes only for bottom left plot of group (a 'group' is an analysis of identical markers).

☒ All plots are contour plots with outliers or pseudocolor plots.

☒ A numerical value for number of cells or percentage (with statistics) is provided.

Methodology

Sample preparation	The source and preparation of blood samples and cell lines is presented in detail in the Method section.
Instrument	Cell sorting was performed using a BD FACSARIA III cell sorter (BD Biosciences) equipped with four lasers (a 405 nm laser, a 488 nm laser, a 561 nm laser and a 640 nm laser). The system was run with 20 psi pressure, a 100 µm nozzle and the yield precision mode. Acquisition of immunofluorescence data was performed with LSR Fortessa cytometer (BD Biosciences) equipped with BD FACS Diva software.
Software	Data were analyzed using the Kaluza Software (Beckman Coulter).
Cell population abundance	Single cells and pools of 2 and 10 single cells were sorted.
Gating strategy	Hoechst 33342-positive elements were first gated. The second gate enabled selection of CD45-APC negative events. CD45-APC-/CK-PE+/ALK-AF488-or+ circulating tumor cells were sorted and collected.

☒ Tick this box to confirm that a figure exemplifying the gating strategy is provided in the Supplementary Information.
